# Salivary α-amylase activity is associated with cardiometabolic and inflammatory biomarkers in overweight/obese, non-diabetic Qatari women

**DOI:** 10.3389/fendo.2024.1348853

**Published:** 2024-03-18

**Authors:** Neyla S. Al Akl, Olfa Khalifa, Mohammad Habibullah, Abdelilah Arredouani

**Affiliations:** ^1^ Diabetes Research Center, Qatar Biomedical Research Institute (QBRI), Hamad Bin Khalifa University (HBKU), Qatar Foundation, Doha, Qatar; ^2^ College of Medicine, Qatar University, Doha, Qatar; ^3^ College of Health and Life Sciences, Hamad Bin Khalifa University (HBKU), Qatar Foundation, Doha, Qatar

**Keywords:** salivary α-amylase activity, obesity, cardiometabolic risk, inflammation, cardiovascular disease

## Abstract

**Introduction:**

Obesity, prevalent in approximately 80% of Qatar’s adult population, increases the risk of complications like type 2 diabetes and cardiovascular diseases. Predictive biomarkers are crucial for preventive strategies. Salivary α-amylase activity (sAAa) inversely correlates with obesity and insulin resistance in adults and children. However, the connection between sAAa and cardiometabolic risk factors or chronic low-grade inflammation markers remains unclear. This study explores the association between serum sAAa and adiposity markers related to cardiovascular diseases, as well as markers indicative of chronic low-grade inflammation.

**Methods:**

Serum samples and clinical data of 1500 adult, non-diabetic, Overweight/Obese participants were obtained from Qatar Biobank (QBB). We quantified sAAa and C reactive protein (CRP) levels with an autoanalyzer. Cytokines, adipokines, and adiponectin of a subset of 228 samples were quantified using a bead-based multiplex assay. The associations between the sAAa and the adiposity indices and low-grade inflammatory protein CRP and multiple cytokines were assessed using Pearson’s correlation and adjusted linear regression.

**Results:**

The mean age of the participants was 36 ± 10 years for both sexes of which 76.6% are women. Our analysis revealed a significant linear association between sAAa and adiposity-associated biomarkers, including body mass index β -0.032 [95% CI -0.049 to -0.05], waist circumference β -0.05 [95% CI -0.09 to -0.02], hip circumference β -0.052 [95% CI -0.087 to -0.017], and HDL β 0.002 [95% CI 0.001 to 0.004], albeit only in women. Additionally, sAAa demonstrated a significant positive association with adiponectin β 0.007 [95% CI 0.001 to 0.01]while concurrently displaying significant negative associations with CRP β -0.02 [95% CI -0.044 to -0.0001], TNF-α β -0.105 [95% CI -0.207 to -0.004], IL-6 β [95% CI -0.39 -0.75 to -0.04], and ghrelin β -5.95 [95% CI -11.71 to -0.20], specifically within the female population.

**Conclusion:**

Our findings delineate significant associations between sAAa and markers indicative of cardiovascular disease risk and inflammation among overweight/obese adult Qatari females. Subsequent investigations are warranted to elucidate the nuances of these gender-specific associations comprehensively.

## Introduction

Ectopic lipid accumulation refers to the deposition of adipose tissue within organs not primarily designated for lipid storage, including the endothelium, liver, and skeletal musculature. This phenomenon is triggered by excessive caloric intake in obesogenic environments, leading to the development of cardiometabolic diseases (1). Obesity, a multifaceted malady, plays a central role in the etiology of numerous afflictions, notably cardiometabolic disorders, persistent low-grade inflammation, insulin resistance (IR), and type 2 diabetes (T2D), among others (2). According to the World Health Organization’s 2022 report, the global population afflicted with obesity surpasses one billion, with an estimated surge of 167 million individuals anticipated by 2025, aligning with the persistent proliferation of obesity-promoting environments (3). Notably, obesity is the major driver for the rising T2D rates, as it heightens its incidence by approximately 7.2-fold (4, 5).

Qatar stands out among Middle Eastern nations in its heightened prevalence of obesity (BMI ≥ 30 kg/m^2^) and overweight (BMI between 25 and 29.9 kg/m^2^), affecting roughly 80% of the adult population (6). This demographic landscape positions overweight/obesity (Ow/Ob) as a principal catalyst for an array of metabolic aberrations, including T2D, non-alcoholic fatty liver disease (NAFLD), dyslipidemia, hypertension, and cardiovascular pathologies (7, 8).

Advancements in identifying reliable biomarkers for predicting obesity are pivotal in formulating effective preventive and management strategies. Salivary α-amylase (sAA), an enzyme instrumental in initiating starch digestion within the oral cavity (9), is encoded by the AMY1 gene. The copy number (CN of the AMY1 gene demonstrates a positive correlation with sAA protein levels and enzymatic activity (10–13). Numerous investigations have linked sAA activity (sAAa) to metabolic disorders, including obesity (14–16), diabetes (17) and IR (18). Our prior research has established an association between elevated sAAa and diminished odds of obesity in the adult population of Qatar (19) and a reduced likelihood of diabetes in Qatari adult women (20).

Despite extensive research investigating the association between sAAa or AMY1 CN and traits related to obesity and glucose metabolism, there was no emphasis on high-risk populations, particularly those characterized by overweight or obesity. Moreover, the intricate nexus between sAAa and other pivotal biomarkers of cardiometabolic risk, such as chronic low-grade inflammation, remains an unexplored avenue in existing scientific literature.

In this study, we aimed to investigate the correlation between sAAa and cardiometabolic risk factors using cardiovascular parameters and chronic low-grade inflammation markers. This examination was conducted within a cohort comprising high-risk Qatari adults characterized by overweight or obesity and otherwise in good health. Furthermore, given the reported differences between men and women regarding sAAa and its relationship with obesity and its comorbidities, we also tested for gender differences.

## Methods

### Study participants

**Figure 1:**
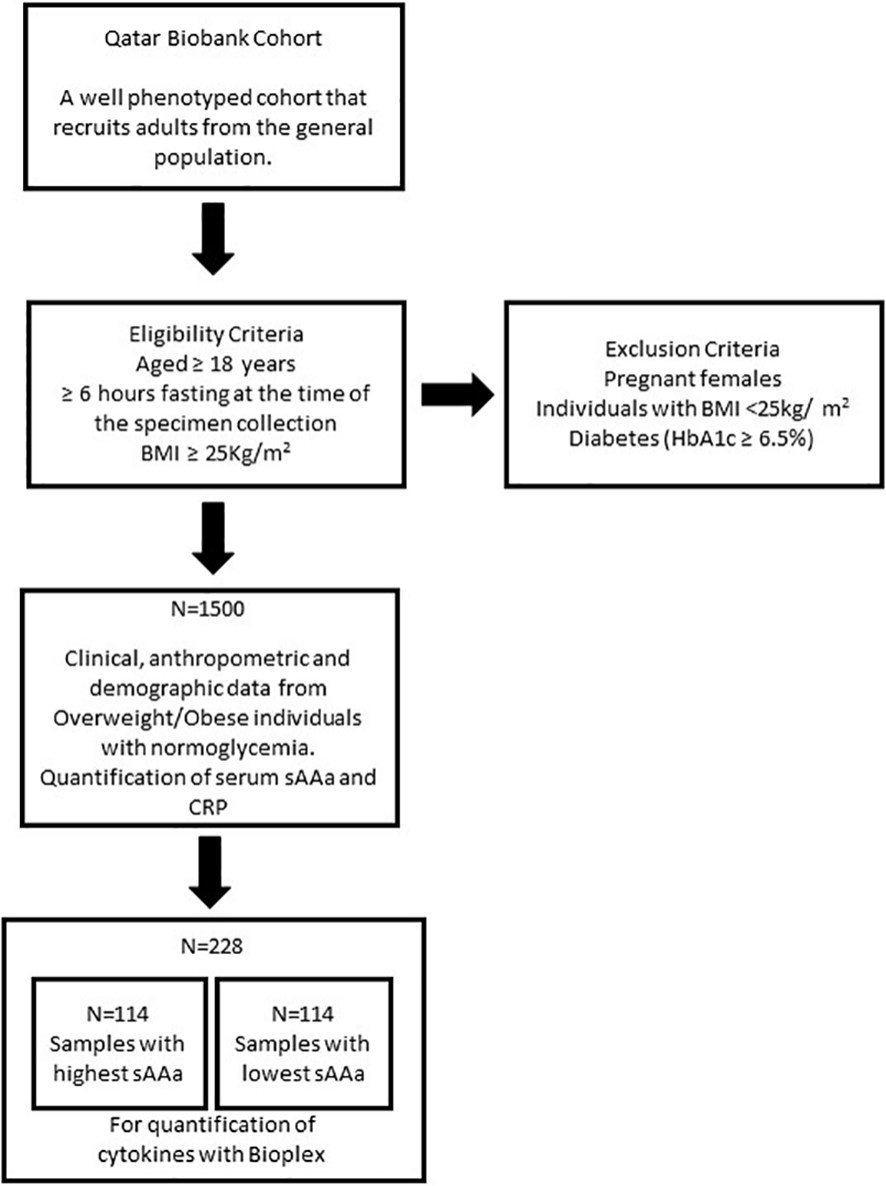
Flow Chart of the study design.

In this cross-sectional investigation, we used baseline clinical, anthropometric, and demographic data from 1500 Ow/Ob individuals with normoglycemia. Serum samples were collected from all participants recruited through the auspices of the Qatar Biobank (QBB) (6). The study’s inclusion criteria encompassed individuals aged 18 years or older who had fasted for a minimum of 6 hours at the time of specimen collection and exhibited a Body Mass Index (BMI) of ≥25 kg/m². Pregnant females, individuals with a normal BMI (<25kg/m^2^), and those presenting with diabetes (as indicated by HbA1c ≥6.5%) were excluded from the study (**Figure 1**). The participation of individuals was contingent upon the provision of informed consent, which authorized the collection and utilization of their data and biological specimens for research purposes, as facilitated by the QBB. Ethical approval for this study was obtained from both the institutional review boards at the QBB (IRB number: Ex-2017-RES-ACC-0054-0018) and the Qatar Biomedical Research Institute (QBRI) (IRB2020-12-052).

### Anthropometric and clinical measures

The central laboratory in Hamad Medical Corporation in Doha carried out all clinical measurements; Fating plasma glucose (FPG), Hemoglobin A1c (HbA1c), triglyceride (TG), total cholesterol (TC), low-density lipid cholesterol (LDL-C), and high-density lipid cholesterol (HDL-C); with an automated biochemical analyzer Body composition was determined by Bioimpedance analysis (Tanita). BMI was calculated as weight in kilograms divided by height in meters squared (kg/m^2^). Fat mass index (FMI) was calculated by dividing fat mass in kilograms by height in meters squared (kg/m^2^). Body adiposity index (BAI) was calculated using the formula: BAI = [((hip circumference (cm)/(height (m)1.5))] − 18 (21). Triglyceride glucose (TyG)-related parameters (TyG), TyG–Body mass index (TyG-BMI), TyG-WC, and TyG-WHTR were calculated using the formulas from Al Akl et al. (22).

### Quantification of the sAAa in serum

The quantification of sAAa was carried out in serum samples using an enzymatic colorimetric assay performed with an autoanalyzer (ARCHITECT c4000; kits #6K22-30 and #7D58-21; ABBOTT Laboratories, Bluff, Illinois, USA). The procedure involved two distinct reactions to assess the enzymatic activities of total α-amylase (tAA) and pancreatic α-amylase (pAA). The determination of sAAa was derived by subtracting the pAA activity from the tAA activity. This assay was externally contracted to Micro Health Laboratories, a private medical laboratory based in Doha (https://www.microhealthcare.com).

### Quantification of circulating serum cytokine, chemokine, and metabolic proteins

The levels of C-reactive protein were assessed in the total cohort. This quantification was conducted utilizing an autoanalyzer (Abbott Architect Platform, Fisher Scientific, USA) at Micro-Health Laboratories in Doha, Qatar.

In a subset of 228 participants, 114 with the highest levels of sAAa and 114 with the lowest levels, serum cytokines and adipokines were quantified by a bead-based multiplex assay (Bio-Plex 3D system, USA), following the established manufacturer’s protocols. Adiponectin and adipsin levels were determined using the Bioplex Pro Human Diabetes Panel (Biorad, Cat# 171A7002M, USA). In contrast, tumor necrosis factor-alpha (TNF-α), interleukin (IL)-6, IL-1β, and monocyte chemoattractant protein-1 (MCP-1) levels were quantified using the Bio-Plex Pro Human Cytokine Grp I Panel 17-plex (Bio-Rad Cat # M5000031YV, USA). Additionally, ghrelin, leptin, glucagon-like peptide 1 (GLP1), gastric inhibitory polypeptide (GIP), resistin, and visfatin levels were assessed using the Bio-Plex Pro Human Diabetes Panel 10-plex (Biorad, Cat#171A7001M, USA). All samples underwent duplicate analysis, and the manufacturer-supplied cytokine standards were concurrently run on each plate. Acquisition parameters were established as follows: MagPlex Beads for gating, 50 μL sample volume, and acquisition of 50 events per bead. Data acquisition was executed utilizing the Bioplex-manager software from Biorad (Biorad, CA, USA), and concentration values were derived compared to a standard curve.

### Statistical analyses

All statistical analyses were carried out with Stata/IC 16.1 software (http://www.stata.com). Normality was inspected, and appropriate transformation was applied in non-normal distribution. Variables with outliers were winsorized using the winsor 2 command in Stata. Missing values were imputed using multiple imputations by chains equations in Stata, and variables with more than 20% missing values were excluded from the analysis. Descriptive statistics were used to present the data mean and standard deviation. Independent samples t-test was used for the comparison of continuous variables between groups. For categorical variables, the chi-square test was used. Correlations were examined with the Pearson coefficient. Simple or multivariate linear regression was used to investigate the association between continuous variables and reported as β-coefficient for quantification. A p-value <0.05 was considered statistically significant.

## Results

### Baseline characteristics of study participants

**Figure 2:**
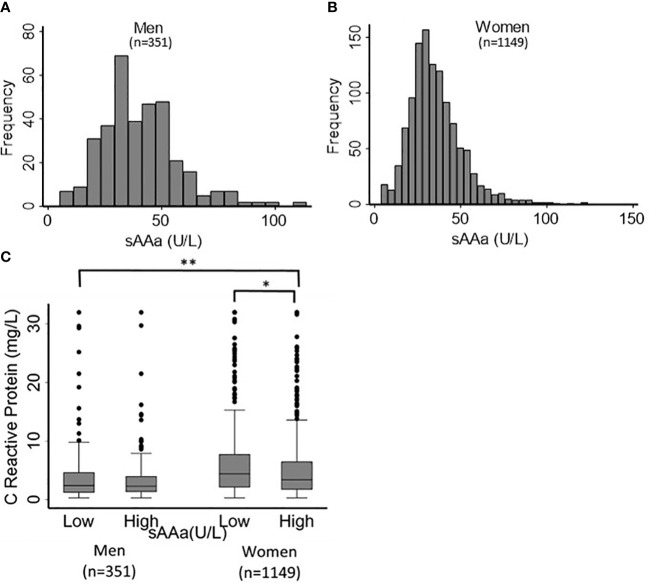
Distribution of sAAa and CRP across participants. Distribution of sAAa concentrations in men **(A)** and women **(B)**. Gender distribution of CRP levels in high and low sAAa groups **(C)**. *and ** indicate P Value <0.05 and <0.001. respectively.

The baseline characteristics of the study participants (76.6% women) are shown in **Table 1**. The mean age was 36 ± 10 years for both sexes. Most adiposity indices, including waist circumference (WC) and hip circumference (HC), waist-to-hip ratio (WHR), body fat percentage (BF%), FMI, BAI, and body weight (BW), were significantly different between men and women. The sAAa ranged from 3.7 to 123.75 U/L (**Figures 2A, B**). Women had a significantly higher low-grade inflammatory marker C-reactive protein (CRP) (**Figure 2C**). The components of the lipid profile, including TC), High-density HDL, TG, and LDL were also significantly different between men and women.

**Table 1:** Baseline characteristics of the participants based on gender.

	Male (n=351) (23.4%)	Female (n=1149) (76.6%)	p-value
sAAa (U/L)	41 ( ± 17)	36 ( ± 16)	<0.001
Age (years)	36 ( ± 10)	36 ( ± 10)	0.81
BMI (Kg/m^2^)	30 ( ± 4)	31 ( ± 5)	0.006
Waist (cm)	95 ( ± 10)	85 ( ± 10)	<0.001
Hip (cm)	108 ( ± 10)	112 ( ± 10)	<0.001
WHR	1 ( ± 0)	1 ( ± 0)	<0.001
Fat Mass (kg)	34 ( ± 10)	34 ( ± 9)	0.70
BF (%)	39 ( ± 13)	45 ( ± 14)	<0.001
FMI (Kg/m^2^)	11.5 ( ± 3.6)	13.7 ( ± 3.9)	<0.001
VAT (kg)	4 ( ± 43)	3 ( ± 26)	0.50
BAI	30 ( ± 4)	38 ( ± 5)	<0.001
BW (Kg)	90 ( ± 15)	78 ( ± 13)	<0.001
FPG (mmol/L)	4.89 ( ± 0)	4.8 ( ± 0)	0.0049
HBA1C %	5 ( ± 0)	5 ( ± 0)	0.53
TC (mmol/L)	5.08 ( ± 0.88)	4.88 ( ± 0.85)	<0.001
HDL (mmol/L)	1 ( ± 0)	2 ( ± 0)	<0.001
TG (mmol/L)	1 ( ± 1)	1 ( ± 0)	<0.001
LDL (mmol/L)	3 ( ± 1)	3 ( ± 1)	<0.001
CRP (mg/L)	2 (1-4)	4 (2-7)	<0.001

Values are reported as Mean ± SD or Median (inter quartile range). sAAa, Salivary a-Amylase activity; BMI, Body Mass Index; WHR, Waist to Hip Ratio; BF, Body Fat percentage; FMI, Fat Mass Index; VAT, Visceral Adiposity Tissue; Body Adiposity Index; BW, Body weight; FPG, Fasting plasma glucose; Tc, Total cholesterol; TG, triglycerides; HDL, High density lipoprotein; LDL, Low density lipoprotein; CRP, c-reactive protein. P Value <0.05 is considered statistically significant.

### Differences in demographic, clinical, and biochemical characteristics between low and high sAAa subjects

Due to the substantial gender-linked variations evident in numerous adiposity indicators and the mean sAAa levels, as delineated in **Table 1**, gender-stratified analysis was due to explore the association between various adiposity metrics and sAAa levels, as presented in **Table 2**. By employing the median sAAa values for males and females, we partitioned the samples into two distinct categories: low and high sAAa groups. Specifically, males with sAAa levels below 39 U/L were categorized as having low sAAa, while those at or above 39 U/L were grouped as high sAAa. For females, the corresponding threshold was set at 33 U/L.

**Table 2:** Gender-stratified clinical and biochemical characteristics of participants based on salivary amylase subgroup.

	Male			Female		
	Low sAAa (n=176)	High sAAa (n=175)	p-value	Low sAAa (n=576)	High sAAa (n=573)	p-value
**sAAa (U/L)**	28 ( ± 7)	54 ( ± 14)	<0.001	24 ( ± 7)	47 ( ± 14)	<0.001
**Age (years)**	36 ( ± 10)	37 ( ± 10)	0.12	36 ( ± 9)	37 ( ± 11)	0.078
**BMI (Kg/m2)**	30 ( ± 5)	30 ( ± 4)	0.70	32 ( ± 5)	31 ( ± 5)	0.002
**Waist (cm)**	95 ( ± 11)	95 ( ± 9)	0.96	85 ( ± 10)	84 ( ± 10)	0.030
**Hip (cm)**	108 ( ± 10)	108 ( ± 9)	0.70	113 ( ± 10)	111 ( ± 9)	0.002
**WHR**	1 ( ± 0)	1 ( ± 0)	0.64	1 ( ± 0)	1 ( ± 0)	0.79
**Fat Mass (kg)**	35 ( ± 11)	33 ( ± 10)	0.19	34 ( ± 9)	34 ( ± 10)	0.92
**BF (%)**	39 ( ± 13)	38 ( ± 13)	0.40	46 ( ± 15)	44 ( ± 13)	0.042
**FMI**	12 ( ± 4)	11 ( ± 3)	0.65	13 ( ± 4)	13 ( ± 4)	0.6
**VAT (kg)**	7 ( ± 61)	2 ( ± 1)	0.32	2 ( ± 1)	4 ( ± 37)	0.15
**BAI**	30 ( ± 5)	30 ( ± 4)	0.98	39 ( ± 5)	38 ( ± 5)	0.019
**BW (Kg)**	90 ( ± 16)	89 ( ± 14)	0.51	79 ( ± 13)	77 ( ± 13)	0.001
**FPG (mmol/L)**	5 ( ± 0)	5 ( ± 1)	0.52	5 ( ± 0)	5 ( ± 0)	0.10
**HBA1C %**	5 ( ± 0)	5 ( ± 0)	0.82	5 ( ± 0)	5 ( ± 0)	0.52
**TC (mmol/L)**	5 ( ± 1)	5 ( ± 1)	0.77	5 ( ± 1)	5 ( ± 1)	0.13
**HDL (mmol/L)**	1 ( ± 0)	1 ( ± 0)	0.23	2 ( ± 0)	2 ( ± 0)	0.007
**TG (mmol/L)**	3 ( ± 1)	3 ( ± 1)	0.80	3 ( ± 1)	3 ( ± 1)	0.53
**LDL (mmol/L)**	1 ( ± 1)	1 ( ± 1)	0.33	1 ( ± 0)	1 ( ± 0)	0.19
**CRP (mg/L)**	4 ( ± 7)	4 ( ± 11)	1.00	6 ( ± 7)	5 ( ± 6)	0.021

All values are reported as Mean ± SD. sAAa, Salivary a-Amylase activity; BMI, Body Mass Index; WHR, Waist to Hip Ratio; BF, Body Fat percentage; FMI, Fat Mass Index; VAT, Visceral Adiposity Tissue; Body Adiposity Index; BW, Body weight; FPG, Fasting plasma glucose; Tc, Total cholesterol; TG, triglycerides; HDL, High density lipoprotein; LDL, Low density lipoprotein; CRP, c-reactive protein. P Value <0.05 is considered statistically significant.

Notable disparities emerged for WC, HC, BF%, BAI, and BW among the adiposity parameters scrutinized. These adiposity parameters exhibited significantly elevated values in females with low versus high sAAa. Conversely, no notable disparities were observed among the two male sAAa subgroups. Likewise, in females, a statistically significant elevation in CRP levels was evident among individuals with low sAAa compared to those with high sAAa (p=0.02). Conversely, no discernible distinction in CRP levels was observed between the male subgroups.

### Relationship of sAAa with adiposity and low-grade inflammatory markers in OW/Ob participants

**Figure 3:**
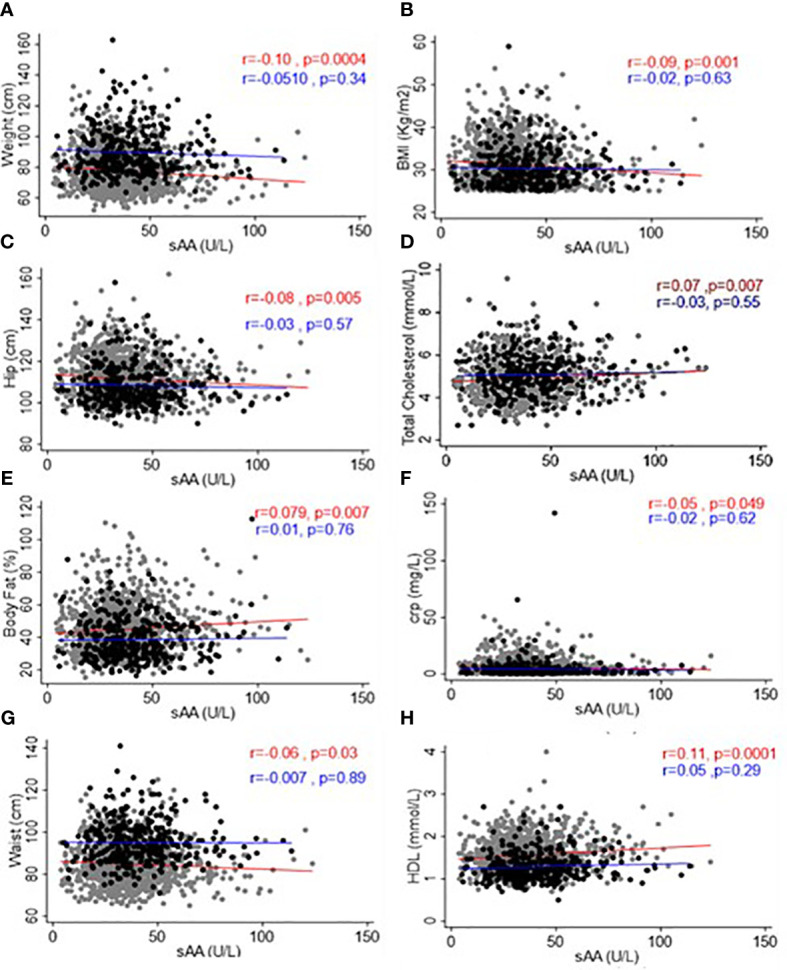
Scatter plots and best-fit lines depicting correlations between sAAa and different adiposity markers, lipids, and CRP. Weight **(A)**, BMI **(B)**, Hip Circumference **(C)**, Total Cholesterol **(D)**, Body fat **(E)**, CRP **(F)**, Waist Circumference **(G)**, and HDL **(H)** in men and women. Gray circles and red lines represent women samples, and black circles and blue lines are for men.

To examine the relationships between sAAa and critical adiposity indicators and CRP levels within our cohort of Ow/Ob individuals, we conducted a rigorous investigation of the corresponding correlations, as illustrated in **Figure 3**. Our analysis revealed noteworthy findings. In the case of female participants, we observed a notable and statistically significant inverse correlation between sAAa and various adiposity markers. Specifically, we noted weak negative but significant associations between sAAa and weight (r = -0.1, p < 0.001), BMI (r = -0.09, p < 0.05), WC (r = -0.06, p < 0.05), and hip circumference (r = -0.08, p < 0.05). The CRP levels were also negatively associated with sAAa (r = -0.05, p < 0.05). Furthermore, in females only, we discerned noteworthy positive correlations of statistical significance between sAAa levels and total cholesterol (r = 0.07, p = 0.007) and HDL (r = 0.11, p = 0.0001). Our analysis yielded neither positive nor negative statistically significant correlations among male participants.

Considering the statistically significant associations established through Pearson’s correlation analysis, we conducted a subsequent linear regression analysis with age adjustment to delve deeper into these associations within both genders. As delineated in **Table 3**, our investigation unveiled age-adjusted associations of statistical significance but in female participants only. These associations manifested as positive or negative correlations between sAAa and a spectrum of adiposity markers, including BMI, weight, WC, HC, and body fat percentage (BF%). Furthermore, these age-adjusted associations extended to lipid parameters, specifically total cholesterol and HDL, as well as the inflammatory marker CRP.

**Table 3:** Linear regression adjusted for age examining the association between sAAa and different adiposity markers and low-grade inflammatory marker CRP.

Variables	Male (n=351) β (95%CI)	Female (n=1149) β (95%CI)
**BMI (kg/m2)**	-0.002 (-0.029 to 0.025)	-0.032 (-0.049 to -0.015) ***
**Weight (kg)**	-0.02 (-0.11 to 0.07)	-0.09 (-0.14 to -0.04) ***
**Waist (cm)**	-0.009 (-0.007 to 0.05)	-0.05 (-0.09 to -0.02) **
**Hip (cm)**	0.001 (-0.056 to 0.059)	-0.052 (-0.087 to -0.017) **
**BF %**	0.006 (-0.073to 0.085)	0.072 (0.02 to 0.12) *
**TC (mmol/L)**	-0.001 (-0.006 to 0.005)	0.003 (0.0001 to 0.0058) *
**HDL (mmol/L)**	0.001 (-0.001 to 0.003)	0.002 (0.001 to 0.004) ***
**CRP (mg/L)**	-0.01 (-0.06 to 0.04)	-0.02 (-0.044 to -0.0001) *

BMI, Body Mass Index; BF%, Body fat %; TC, Total Cholesterol; HDL, High-Density Lipoprotein; CRP, C reactive Protein *, **, *** significant at p <5%, p<1% and p<0.01% respectively.

We used triglyceride indices as analytical tools for our linear regression analysis to further elucidate the relationship between sAAa and cardiovascular risk with greater precision. The formulas utilized to compute these indices were as in our previous publication (22). The disparities in these indices between male and female subjects are presented in **Table 4**.

**Table 4:** Obesity and triglycerides indices in participants.

	Male (n=351)	Female (n=1149)	p-value
**TY-G**	8.35( ± 0.50)	8.2( ± 0.42)	<0.001
**TYG-BMI**	254( ± 39)	256( ± 44)	0.45
**TYG-WC**	795( ± 98)	696( ± 99)	<0.001
**TYG-WHTR**	4.6( ± 0.6)	4.4( ± 0.6)	<0.001

WHTR, Waist to Height ratio; TyG, Triglyceride-glucose; TyG-BMI, Triglyceride-glucose-BMI; TyG-WC, Triglyceride-glucose-waist circumference; TyG-WHTR, Triglyceride-glucose-waist-to-height ratio.

Except for the TyG-BMI, the Triglyceride-Glucose (TyG), the Triglyceride-Glucose-Waist Circumference (TyG-WC), and the Triglyceride-Glucose-Waist-to-Height Ratio (TyG-WHTR) indices were significantly different between genders. Age-adjusted regression analysis unveiled significant negative associations between sAAa and TyG-BMI, TyG-WC, and TyG-WHTR, but only in females (**Table 5**).

**Table 5:** Linear regression adjusted for age examining the association between sAAa and Triglyceride indices.

Variables	Male (n=351) β (95%CI)	Female (n=1149) β (95%CI)
TyG	-0.001 (-0.004 to 0.001)	-0.001 (-0.002 to 0.002)
TyG-BMI	-0.48 (-0.28 to 0.19)	-0.303 (-0.45 to -0.14) ***
TyG-WC	-0.19 (-0.78 to 0.39)	-0.525 (-0.86 to -0.18) **
TyG-WHTR	-0.001 (-0.004 to 0.002)	-0.003 (-0.005 to -0.001) **

TyG:,Triglyceride-glucose; TyG-BMI, Triglyceride-glucose-BMI; TyG-WC, Triglyceride-glucose-waist circumference; TyG-WHTR, Triglyceride-glucose-waist-to-height ratio. *, **, *** significant at p <5%, p<1% and p<0.01% respectively.

### Associations of sAAa with inflammatory cytokines and gut hormones in Ow/Ob participants

To investigate the association between sAAa and inflammatory cytokines, we ran a linear regression adjusted for age in men and women, as shown in **Table 6**. In women, we observed a significant inverse association of sAAa with the pro-inflammatory cytokines IL-6 and TNF- α, while the association with adiponectin was significant and positive. Furthermore, ghrelin, known as the hunger hormone (23), showed a significant inverse relationship with sAAa in obese female participants. GLP1 and GIP were also investigated; however, their concentrations were below the detection limit in over 40% of the samples.

**Table 6:** Age-adjusted Linear regression examining the association between sAAa and different inflammatory markers.

Variables	Male (n=46); β (95%CI)	Female (n=182); β (95%CI)
IL-6	-0.39 (-0.93 to 0.13)	-0.39 (-0.75 to -0.04)*
Adiponectin	-0.004 (-0.015 to 0.006)	0.007 (0.001 to 0.01)*
TNF-α	-0.14 (-0.34 to 0.05)	-0.105 (-0.207 to -0.004)*
Ghrelin	-6.57 (-13.27 to 0.12)	-5.95 (-11.71 to -0.20)*

IL-6, Interleukin 6; TNF-α, Tumor necrosis factor alpha *significant at p <5%.

## Discussion

This cross-sectional investigation elucidated associations between sAAa and various cardiometabolic and inflammatory biomarkers in Ow/Ob Qatari women. Among these biomarkers were adiposity metrics, including BMI, BW, WC, HC, and triglyceride-derived indices TyG-BMI, TyG-WC, and TyG-WHTR. Females exhibiting elevated sAAa levels demonstrated statistically significant reductions in these biomarkers. Also, this cohort’s heightened sAAa levels correlated with statistically significant decreases in the inflammatory marker CRP and an increase in the anti-inflammatory adipokine adiponectin. These findings were further characterized by a negative correlation between sAAa and circulatory levels of the pro-inflammatory cytokines IL-6 and TNF-α. Furthermore, a reduction in ghrelin, the gastric hormone, was observed in these females.

The results of our investigation align with findings from the literature regarding the instrumental role of gold-standard adiposity metrics in assessing CVD risk in Ow/Ob individuals (24–27). Indeed, data demonstrated significant inverse correlations of sAAa with key adiposity indices, including BMI, BW, WC, and HC in the context of Ow/Ob women. Moreover, previous studies have underscored the association of an elevated TyG index with the onset and prognostication of CVD (28). The present study unveiled significant age-adjusted inverse relationships between sAAa and TyG-derived indices, specifically TyG-BMI, TyG-WC, and TyG WHTR, in Ow/Ob women. These observations posit diminished sAAa levels as a potential indicator for heightened CVD risk. Within the lipids spectrum, our study revealed a positive correlation between sAAa and HDL levels in women. HDL, known for its anti-inflammatory properties, exerts a protective influence against CVD by orchestrating cholesterol efflux from tissues (29). The positive association between the two biomarkers confirms the role of high sAAa as a potential indicator of CVD. These findings align with the existing literature, where sAAa has demonstrated a potential antidiabetic and anti-CVD effect (30).

The basis of the link between obesity and cardiovascular disease can be attributed to the influence of different hormones and circulating factors such as adipokines on the chronic inflammation seen in obese subjects (31). It was reported that obese individuals have altered circulatory levels of inflammatory adipokines and cytokines, such as IL-6, TNFα, CRP, IL-18, resistin, visfatin and adiponectin (32–34). Adiponectin plays a conspicuous role in cardiovascular diseases, T2D, and metabolic syndrome (35, 36). It also exerts significant actions on the innate and adaptive immune systems, and is known for its anti-inflammatory effects by suppressing the production of pro-inflammatory cytokines such as TNF-α, IL-6 and CRP (37). Our finding that elevated sAAa is significantly and positively associated with adiponectin levels in obese women along with the significant negative association with decreased levels in the pro-inflammatory proteins TNF- α, IL-6 and CRP in our study indicate that sAAa could potentially be a good marker for the inflammatory status of the individual. In fact, the secretion of IL-6 from adipose tissue is presumed to cause elevation in CRP levels with increase in body fat (32) and CRP is a well-known marker of systemic and cardiac-related inflammation that is widely used to predict cardiovascular risk (38). Furthermore, in obesogenic environment, ghrelin is decreased (39) (40) and adipokines are stimulated. Indeed, sAAa was positively associated with adiponectin and negatively associated with ghrelin in our study.

The gender-dependent variance observed in the association between sAAa and cardiometabolic indices needs to be investigated further but could be attributed to differences in stress and anxiety observed between men and women and as such affecting levels of inflammatory cytokines (30, 41). Other non-traditional risk factors specific to women, such as excess weight gain during pregnancy, preeclampsia, gestational diabetes, preterm delivery, and menopause, could also be implicated (42). Our one gender sided association concurs with the finding of Ikeda et al, depicting an association between sAAa and higher risk of CVD in women (30).

Our results stand out as they stem from a substantial sample, concentrating solely on individuals with overweight/obesity, with the deliberate exclusion of diabetes as a predisposing factor for cardiovascular diseases (CVD).

The major limitation of the present study is the cross-sectional design, which limits our ability to draw causal inferences. Although we adjusted for age, various residual confounding factors such as genetic factors, environmental factors, smoking status, exercising, alcohol consumption, and diet may influence the cardiometabolic disease causality cascade. Moreover, the study is limited by the lack of information regarding medical treatments that participants might be undergoing, potentially influencing the levels of factors examined. Additionally, the absence of result validation serves as another limitation, constraining the generalizability of our findings. Nevertheless, our study stands as the pioneer in exploring the relationship between serum sAAa and adiposity markers associated with risk of CVD activity and markers of chronic low-grade inflammation in Qatari overweight/obese adults while accounting for caution when extrapolating these findings to diverse populations.

In conclusion, the well-established connection between obesity, chronic inflammation, and cardiac diseases highlights the urgency of identifying individuals at risk of developing cardiovascular diseases, particularly in the context of the global obesity epidemic. Our findings suggest that salivary α-amylase activity (sAAa), exhibiting associations with adiposity, triglyceride indices, low-grade inflammatory cytokines (CRP), and adiponectin along with its associated inflammatory cytokines, holds promise as a valuable tool for detecting predisposition to cardiovascular diseases in obese women in Qatar. Further analysis, including long-term patient follow-up and the incorporation of environmental and lifestyle data, is warranted to robustly confirm the observed conclusions.

## Data availability statement

Anthropometric, demographic, and clinical data of the participants was obtained from the Qatar Biobank Cohort upon the submission of a proposal and approval by the IRB. We do not have the permission to share the data, and, thus, no permission can be provided. The authors did not receive any special privileges in accessing the data used in the study. Any Researcher can access the data upon the submission of a proposal and approval of the IRB. The data can be accessed by submitting and application through the QBB website (https://researchportal.qatarbiobank.org.qa/login). Researchers need to create an account to be able to submit an application.

## Ethics statement

The studies involving humans were approved by IRB at Qatar Biomedical Research Institute IRB at Qatar Biobank. The studies were conducted in accordance with the local legislation and institutional requirements. The participants provided their written informed consent to participate in this study.

## Author contributions

NA: Formal Analysis, Investigation, Methodology, Validation, Writing – original draft, Writing – review & editing. OK: Methodology, Writing – review & editing. MH: Methodology, Writing – review & editing. AA: Conceptualization, Data curation, Funding acquisition, Resources, Supervision, Writing – review & editing.
